# Overexpression of *HMGA2* promotes tongue cancer metastasis through EMT pathway

**DOI:** 10.1186/s12967-016-0777-0

**Published:** 2016-01-27

**Authors:** Xiao-Peng Zhao, Hong Zhang, Jiu-Yang Jiao, Dong-Xiao Tang, Yu-ling Wu, Chao-Bin Pan

**Affiliations:** Guangdong Provincial Key Laboratory of Malignant Tumor Epigenetics and Gene Regulation, Sun Yat-Sen Memorial Hospital, Sun Yat-Sen University, Guangzhou, China; Department of Oral & Maxillofacial Surgery, Sun Yat-sen Memorial Hospital, Sun Yat-sen University, Guangzhou, China; Guanghua School of Stomatology, Hospital of Stomatology, Sun Yat-sen University, Guangdong Provincial Key Laboratory of Stomatology, Guangzhou, Guangdong China

**Keywords:** HMGA2, TSCC, Metastasis, EMT, Snail

## Abstract

**Background:**

Metastasis to long distance organs is the main reason leading to morality of tongue squamous cell carcinoma (TSCC); however, the molecular mechanisms are still unknown. High mobility group AT-hook 2 (HMGA2) is highly expressed in multiple metastatic carcinomas, in which it contributes to cancer progression, metastasis and poor prognosis by upregulating Snail expression and inducing epithelial mesenchymal transition (EMT). This study focuses on investigating the role and mechanism of regulation of HMGA2 in the metastasis of TSCC.

**Methods:**

HMGA2 mRNA and protein expression were examined in TSCC specimens by quantitative real-time polymerase chain reaction, western blotting and immunohistochemistry (IHC). Western blotting, IHC and immunofluorescence were also used to measure the expression and localization of EMT marker E-Cadherin and Vimentin both in TSCC cells and tissues. Knockdown assay was performed in vitro in TSCC cell lines using small interfering RNAs and the functional assay was carried out to determine the role of HMGA2 in TSCC cell migration and invasion.

**Results:**

TSCC mRNA and protein expression were significantly up-regulated in tumor tissues when compared to adjacent non-tumor tissues, and the overexpression of HMGA2 was closely correlated with lymph nodes metastasis. Clinicopathological analysis indicated that HMGA2 expression was associated with clinical stage (*P* = 0.001), lymph node metastasis (*P* = 0.000), histological differentiation (*P* = 0.002) and survival (*P* = 0.000). Silencing the *HMGA2* expression in Cal27 and UM1 resulted in the inhibition of cell migration and invasion, meanwhile down-regulation of HMGA2 impaired the phenotype of EMT in TSCC cell lines and tissues. The Multivariate survival analysis indicates that HMGA2 can be an independent prognosis biomarker in TSCC.

**Conclusion:**

Our findings demonstrate that HMGA2 promotes TSCC invasion and metastasis; additionally, HMGA2 is an independent prognostic factor which implied that HMGA2 can be a biomarker both for prognosis and therapeutic target of TSCC.

## Background

Tongue squamous cell carcinoma (TSCC) is one of the most common and lethal oral cancer [1, 2], which is characterized by its preferring of lymph node and distant metastasis [3]. Clinical evidences indicate that metastasis is the most important poor prognostic factors for patient diagnosis with TSCC [4]. Despite its significance and the enormous studies accumulated in the past decades on the molecular mechanisms of TSCC progression, little is known about the underlying molecular mechanisms regulating metastatic dissemination.

More and more studies demonstrated that epithelial mesenchymal transition (EMT) is a key process which has been shown to be of critical biological function and significance during embryogenesis and carcinogenesis [5–7]. Increasing evidences have recognized that the epithelial to mesenchymal transition (EMT), a driver of invasion and metastasis of cancer, may play a pivotal role in multiple types of tumor cell metastatic dissemination by endowing cells with a more motile, invasive potential [8–11].

High mobility group 2 (HMGA2) is a chromatin remodeling factor which can change the chromatin architecture to activate or impair the activity of transcriptional enhancers [12]. HMGA2 is highly expressed in most malignant epithelial tumors, including breast cancer [13, 14], colorectal cancer [15], gastric cancer [16], lung cancer [17], melanoma [18], myeloid [19], oral cancer [20], ovarian cancer [21], pancreas cancer [22], pituitary adenomas [23, 24]. HMGA2 overexpression in transgenic mice causes tumorigenesis; however, HMGA2-knockout in mice can severely impair the mice growth and development, leading a nanous shape [25].

Despite the fact that both the HMGA2 and EMT play a significant role in the development and progression of TSCC, the relationship between these factors has not yet been reported in TSCC. In the present study, we demonstrate that overexpression of HMGA2 is closely associated with progression and poorer overall survival in human TSCC, and provide evidence that the expression of HMGA2 can promote the progression of TSCC by upregulating Snail and inducing the EMT.

## Methods

### Patients and tissue samples

A total of 60 human TSCC tissues and 20 adjacent non-tumor tissue samples were examined in this study. The patients were histopathologically and clinically diagnosed at Sun Yat-sen Memorial Hospital, Sun Yat-sen University from 2008 to 2010; the pathological diagnosis was verified for each case. For each case, tumor samples with matched adjacent non-tumor tissue samples were collected during surgical resection and frozen in liquid nitrogen and stored at −80 °C. Sample collection was performed in accordance with the policies of the National Research Ethics Committee and informed consent was obtained from each patient. The clinicopathological features of the patients are summarized in Table 1.

**Table 1:** Clinicopathological parameters and HMGA2, Snail1 expression in 60 primary tongue carcinomas

Parameter	n	HMGA2 staining	*P*	Snail1 staining	*P*
		Positive (%)		Positive (%)	
Age			0.863		0.134
≤55	34	22 (64.7)		17 (50.0)	
>55	26	15 (57.7)		8 (30.8)	
Sex			0.005		0.394
Female	23	9 (39.1)		8 (34.8)	
Male	37	28 (75.7)		17 (45.9)	
T stage			0.074		0.895
T1 + T2	33	17 (63.6)		14 (45.5)	
T3 + T4	27	20 (81.5)		11 (59.3)	
Clinical stage			0.001		0.003
I + II	23	8 (34.8)		4 (17.4)	
III + IV	37	29 (78.4)		21 (56.8)	
N status			0.000		0.000
N^−^	35	14 (40.0)		6 (17.1)	
N^+^	25	23 (92.0)		19 (76.0)	
Histological differentiation			0.002		0.001
Well	24	9 (37.5)		4 (16.7)	
Moderate/poor	36	28 (77.8)		21 (58.3)	
Survival			0.000		0.001
Survival	32	11 (34.4)		7 (21.9)	
Die	28	26 (92.9)		18 (64.3)	

### Cell lines and cell cultures

The human TSCC cell Cal27, SCC9, SCC15, SCC25 and UM1 were used in our study. Cal27, SCC9, SCC15 and SCC25 cell lines were obtained from American Type Culture Collection (ATCC; Manassas, VA, USA) and UM1 was reserved by our lab. Cal27 cells were maintained in DMEM medium (Invitrogen, Carlsbad, CA, USA) supplemented with 10 % fetal bovine serum (FBS) and other cells were cultured in RPMI-1640 medium supplemented with 10 % FBS. For all TSCC cell lines, 1 % penicillin/streptomycin was added to the culture medium and all TSCC cell lines were cultured at 37 °C in a humidified atmosphere containing 5 % CO_2_.

### RNA extraction, reverse transcription and quantitative real-time PCR (qRT-PCR)

For total RNA isolation, tumor specimens were finely minced with scissors and homogenized, then, the total RNA from fresh surgical tongue tissues and TSCC cells were extracted using the TRIzol reagent (Invitrogen, Carlsbad, California, USA) according to the manufacturer’s instructions. Complementary DNA (cDNA) was synthesized with the PrimeScript RT reagent Kit (TaKaRa, Dalian, China) primed with random hexamers. For amplification of HMGA2, reverse transcription PCR was programmed as follows: 95 °C for 2 min, 30 cycles of 94 °C for 30 s, 56 °C for 30 s, 72 °C for 45 s, 72 °C for 10 min, hold at 4 °C. The primer was as followed: HMGA2 forwared: 5′-AAGTTGTTCAGAAGAAGCCTGCTCA-3′; HMGA2 reverse: 5′-TGGAAAGACCATGGCAATACAGAAT-3′. RT-PCR products were analyzed via 2.0 % agarose gel electrophoresis and stained with ethidium bromide for visualization using ultraviolet light. Real-time PCR was performed with LightCycler Real Time PCR System (Roche Diagnostics, Switzerland) and the primer sequences for *HMGA2* was used as followed: (F) 5′-AAAGCAGCTCAAAAGAAA GCA-3′; (R) 5′-TGTTGTGGCCATTTCCTAGGT-3′.

### RNA interference

Short interfering RNA (siRNA) against *HMGA2* and corresponding GFP siRNA (GFP-si) were synthesized and purchased from GenePharma Company (GenePharma, Shanghai, China). The two siRNAs specific against HMGA2 sequences were as followed: HMGA2-siRNA1: CACAACAAGUCGUUCAGAA; and HMGA2-siRNA2: AGAGGCAGACCUAGGAAAU. Transfection was performed in 6-well plates using Lipofectamine 2000 (inviztrogen) following the manufacturer’s instructions. The gene silencing efficiency was detected by western blotting after transfection.

### Western blotting

Equal amounts of protein extracts were separated using 10 % polyacrylamide SDS gels (SDS–PAGE), transferred onto polyvinylidene fluoride (PVDF) membranes (Amersham Pharmacia Biotech) and the membranes were probed with antibody against human HMGA2 (1:1000, Cell Signal Technology, Danvers, MA, USA), E-cadherin, vimentin, snail (1:500, Santa Cruz, Santa Cruz, CA, USA), or GAPDH (1:3000, Proteintech, Chicago, IL, USA), and then with peroxidase-conjugated secondary antibody (1:3000, Proteintech) and the signals were visualised by enhanced chemiluminescence kit (GE, Fairfield, CT, USA) according to the manufacturer’s instructions. Anti-GAPDH antibody (Proteintech) was used as a loading control.

### Modified boyden chamber assay

A total of 1 × 10^5^ cells were plated into the upper chamber of a polycarbonate transwell filter chamber (Corning, New York, NY, USA) and incubated for 10 h. For invasion assay, the upper chamber was coated with Matrigel (R&D, Minneapolis, MN, USA) and incubated for 24 h. The non-invading cells were gently removed with a soft cotton swab, and the cells that had invaded to the bottom chamber were fixed, stained, photographed and counted.

### Immunofluorescence analysis

Cells were seeded on glass coverslips, cultured, fixed and subjected to immunofluorescent analysis by incubation overnight at 4 °C with antibodies against E-cadherin or vimentin (1:100, Santa Cruz, Santa Cruz, CA, USA). After washing several times, the cells were incubated with Alexa Fluor 594-conjugated secondary antibodies (1:500, Invitrogen, USA) for 1 h at room temperature, then the cells were counterstained with DAPI and imaged by confocal laser-scanning microscopy (LSM710, Carl Zeiss, Thornwood, NY).

### Immunohistochemistry

Immunohistochemical analysis was performed to investigate the expression of HMGA2, Snail, E-Cadherin and Vimentin in different grades of human tongue cancer. Briefly, immunohistochemistry (IHC) was performed on the paraffin-embedded human tongue cancer tissue sections. Antigen retrieval was performed in a pressure cooker in citrate solution, pH 6.0, for 15 min, followed by treatment with 3 % hydrogen peroxide for 5 min. Specimens were incubated with antibodies as followed: goat monoclonal antibodies against HMGA2 (1:100, CST), E-cadherin, vimentin, snail (1:100, Santa Cruz, Santa Cruz, CA, USA). For the negative controls, isotype-matched antibodies were applied. The tissue sections were observed under a Zeiss AX10-Imager A1 microscope (Carl Zeiss, Thornwood, NY) and all images were captured using AxioVision 4.7 microscopy software (Carl Zeiss, Thornwood, NY).

### Statistical analysis

Statistical analysis was performed using a SPSS software package (SPSS Standard version 18.0, SPSS Inc). (SPSS, Chicago, IL, USA) Differences between variables were assessed by the χ^2^ test according to Pearson or Fisher’s exact test. For survival analysis, we analysed all patients with TSCC by Kaplane–Meier analysis. A log rank test was used to compare different survival curves. Multivariate survival analysis was performed on all parameters that were found to be significant in univariate analysis using the Cox regression model. Two-tailed Student’s t tests were used to determine statistical significance for all results. *P* < 0.05 was considered to be statistically significant in all cases.

## Results

### HMGA2 expression is up-regulated in TSCC cells lines

**Fig. 1:**
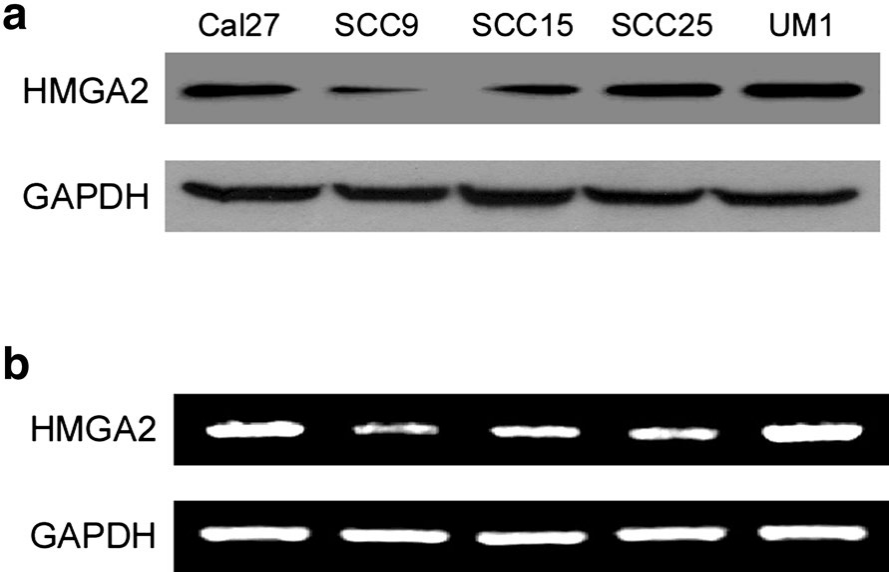
Overexpression of HMGA2 in human tongue cancer cell lines. **a** Western blotting analysis of HMGA2 protein in tongue squamous cell carcinoma (TSCC) cell lines. GAPDH was probed as loading control. **b** Real-time PCR analysis of HMGA2 mRNA expression level in tongue cancer cell lines

Overexpression of HMGA2 has been reported in many kinds of human cancers. Its expression status in tongue cancer, however, remains unknown. To investigate the HMGA2 expression, western blotting analysis were carried out to quantify the expression level of HMGA2 in TSCC cell lines. The results demonstrated that HMGA2 protein was highly expressed in tongue cancer cell lines (Fig. 1a). To confirm if the HMGA2 upregulation was also apparent at the mRNA level, reverse transcription-PCR was performed. As shown in Fig. 1b, in parallel with up-regulation of the HMGA2 protein, the five tongue cancer cell lines unexceptionally showed significantly higher levels of HMGA2 mRNA.

### HMGA2 is overexpressed in primary tongue cancer

**Fig. 2:**
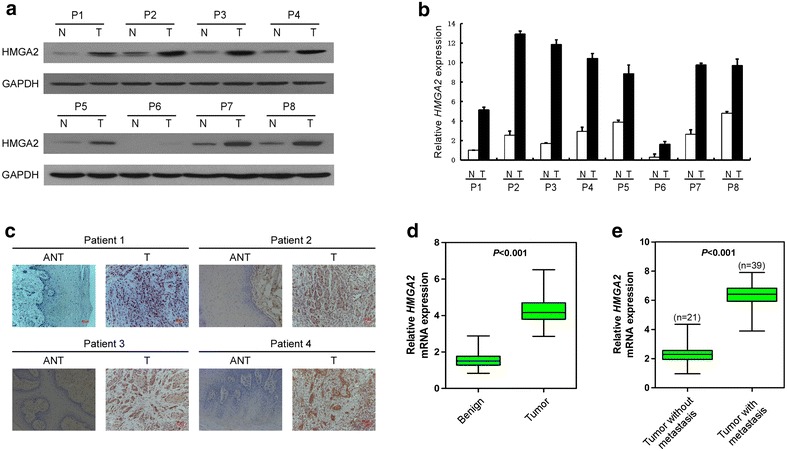
HMGA2 is upregulated in histopathological sections of tongue cancer and high expression of HMGA2 is correlated with tumorigenesis and metastasis. **a** Western blotting analysis of HMGA2 protein in eight human primary tongue cancer (T) and paired adjacent non-tumor tongue tissues (N), with each pair taken from a same patient. **b** Quantitative real time RT-PCR analysis of HMGA2 mRNA from the same eight pairs of tongue cancer and adjacent non-tumor tongue tissues. *Error bars* represent SDs calculated from three parallel experiments. **c** HMGA2 expression levels were up-regulated in primary tongue tumor tissues (T) compared to the paired adjacent non-tumor tongue tissue (N) from the same patient as examined by immunohistochemistry. **d** Expression levels of HMGA2 in 20 paired TSCC and adjacent non-tumor tongue tissues. Alteration of expression is shown as *box plot* presentations and the mean level of HMGA2 expression in TSCCs were significantly higher than that in non-tumor tissues. (*P* < 0.001, independent t test). **e** Expression levels of HMGA2 between 60 TSCCs with and without metastasis. The mean level of HMGA2 expression in TSCCs with metastasis were significantly higher than that in TSCCs without metastasis (*P* < 0.001, independent t test)

To determine whether the up-regulation of HMGA2 in tongue cancer cell lines is clinically correlated with tongue cancer progression, we did western blotting analysis on eight cases of paired primary matched adjacent non-neoplastic tongue tissue and tongue cancer samples. As shown in Fig. 2a, consistent with the former results, HMGA2 was found to be differentially overexpressed in all eight examined human primary tongue cancer samples compared with their matched adjacent non-neoplastic tissues from the same patients. This result is also can be obtained in real-time reverse transcription-PCR (Fig. 2b). Furthermore, this finding is consistent with the results obtained in our immunohistochemical staining analysis in four pairs of primary tongue cancer tissues and adjacent non-neoplastic tissues (Fig. 2c). Additionally, using quantitative real-time PCR, 20 cases of paired primary matched adjacent non-neoplastic tongue tissue and tongue cancer samples were analyzed, most of cases had much higher *HMGA2* expression levels, compared with adjacent non-neoplastic tongue tissues (Fig. 2d). All of these results indicate that HMGA2 was upregulated in tongue cancer cell lines and human tissues.

### Overexpression of HMGA2 was associated with a poor prognostic phenotype of TSCCs

To further investigate the clinicopathological and prognostic significance of HMGA2 levels in patients with TSCC, the levels of HMGA2 in a large cohort of 60 TSCC tissues were examined by qRT-PCR and then verified by IHC. Using qRT-PCR, the correlation between HMGA2 expression and metastatic status was analyzed in 60 TSCC samples. The result showed that, 39/60 (65 %) TSCC had much higher expression of HMGA2, which was significantly associated with a more aggressive tumor phenotype (*P* < 0.001, Fig. 2e). To further confirm the verified the results above, IHC was performed in all the 60 TSCC samples. The median value of all 60 TSCC samples was chosen as the cut-off point for separating tumors with negative expression of HMGA2 from positive expression HMGA2 tumors; thus 37/60 (61.7 %) TSCCs had positive expression of HMGA2, while 23/60 (38.3 %) TSCCs had negative expression of HMGA2 (Table 1). Furthermore, as shown in Table 1, HMGA2 expression strongly correlated with clinical stage (*P* = 0.001), lymph node status (*P* = 0.000), histological differentiation (*P* = 0.002) and survival (*P* = 0.000) in patients with tongue cancer; however, the analysis data indicated that HMGA2 expression was not correlated with age and tumor stage. Taken together, our analyses revealed that the expression of HMGA2 was upregulated during the clinical progression of tongue cancer, indicating that the expression of HMGA2 may promote the progression of tongue cancer.

### Association between HMGA2 expression and patient survival

**Fig. 3:**
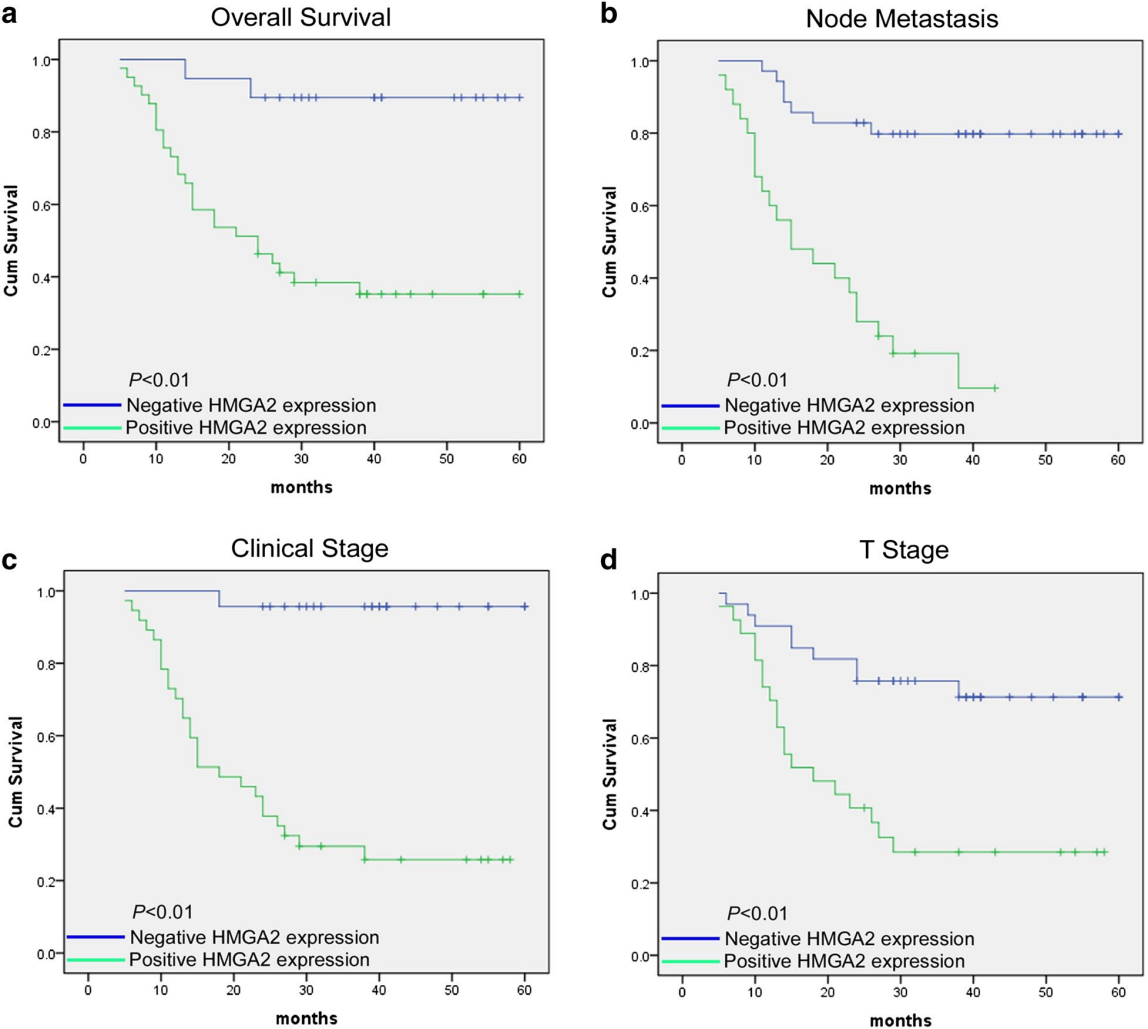
Kaplan–Meier analysis of TSCC patients with positive HMGA2 expression versus those that negative HMGA2 expression. **a** Overall survival rate for cases with positive HMGA2 expression versus that of cases with negative HMGA2 expression; **b** HMGA2 expression status for overall survival rate stratified by lymph node metastasis condition; **c** overall survival rate for cases categorized with clinical stage classification and HMGA2 expression status; **d** HMGA2 expression level for overall survival rate stratified by tumor stage classification

Patient survival analysis presented in Table 1 indicated a clear positive correlation between HMGA2 protein expression level and the overall survival time in tongue cancer patients. The effects of classic clinicopathologic features, including age, gender, clinical stage, tumor stage (T stage), lymph node status (N status), distant metastasis, in conjunction with HMGA2 protein expression, on patient survival, were examined with Kaplan–Meier analysis and the log-rank test. As shown in Fig. 3a, the length of overall survival time varied significantly different between patients with negative and positive HMGA2 expression (*P* < 0.01), with the negative HMGA2 expression group having a longer overall survival time, compared with those with high level expression of HMGA2. In addition, the prognostic value of HMGA2 expression in patient was also evaluated according to the lymph node metastasis, clinical stage and T stage. The analysis results revealed that the patients with tumors exhibiting low expression of HMGA2 have less possibility of lymph node metastasis (Fig. 3b), well prognosis of clinical and tumor stage (Fig. 3c, d).

When univariate and multivariate analyses were done to determine whether HMGA2 expression is an independent prognostic factor of patient outcomes, as Table 2 shows, HMGA2 expression (*P* = 0.042), as well as clinical stage (*P* = 0.028), was recognized as independent prognostic factors. Taken together, our data suggest that HMGA2 might represent a novel and potentially useful independent biomarker for the prognosis of patients with tongue squamous cell carcinoma.

**Table 2:** Univariate and multivariate analysis of factors associated with disease-free survival of patients with TSCC

Variable	N	Univariable			Multivariable		
		HR	95 % CI	*P*	HR	95 % CI	*P*
Age							
≤55	34	1					
>55	26	0.615	0.234–1.617	0.324			
Sex							
Male	37	1			1		
Female	23	0.317	0.128–0.784	0.013	0.790	0.649–2.175	0.649
Differentiation							
Well	24	1			1		
Moderate/poor	36	3.829	1.537–9.538	0.004	2.323	0.760–7.103	0.139
T stage							
T1 + T2	33	1			1		
T3 + T4	27	3.638	1.638–8.082	0.002	1.087	0.397–2.972	0.871
N status							
N^−^	35	1			1		
N^+^	25	7.706	3.206–18.522	0.000	1.501	0.4594–4.908	0.501
Clinical stage							
I + II	23	1			1		
III + IV	37	8.873	3.902–13.623	0.001	13.806	1.333–143.024	0.028
HMAG2 staining							
Negative	23	1			1		
Positive	37	14.779	3.431–63.665	0.000	5.785	1.066–31.391	0.042
Snail staining							
Negative	35	1			1		
Positive	25	3.819	1.738–8.393	0.001	0.976	0.280–3.402	0.970

### Down-regulation HMGA2 expression inhibited tongue cancer cell migration and invasion in vitro

**Fig. 4:**
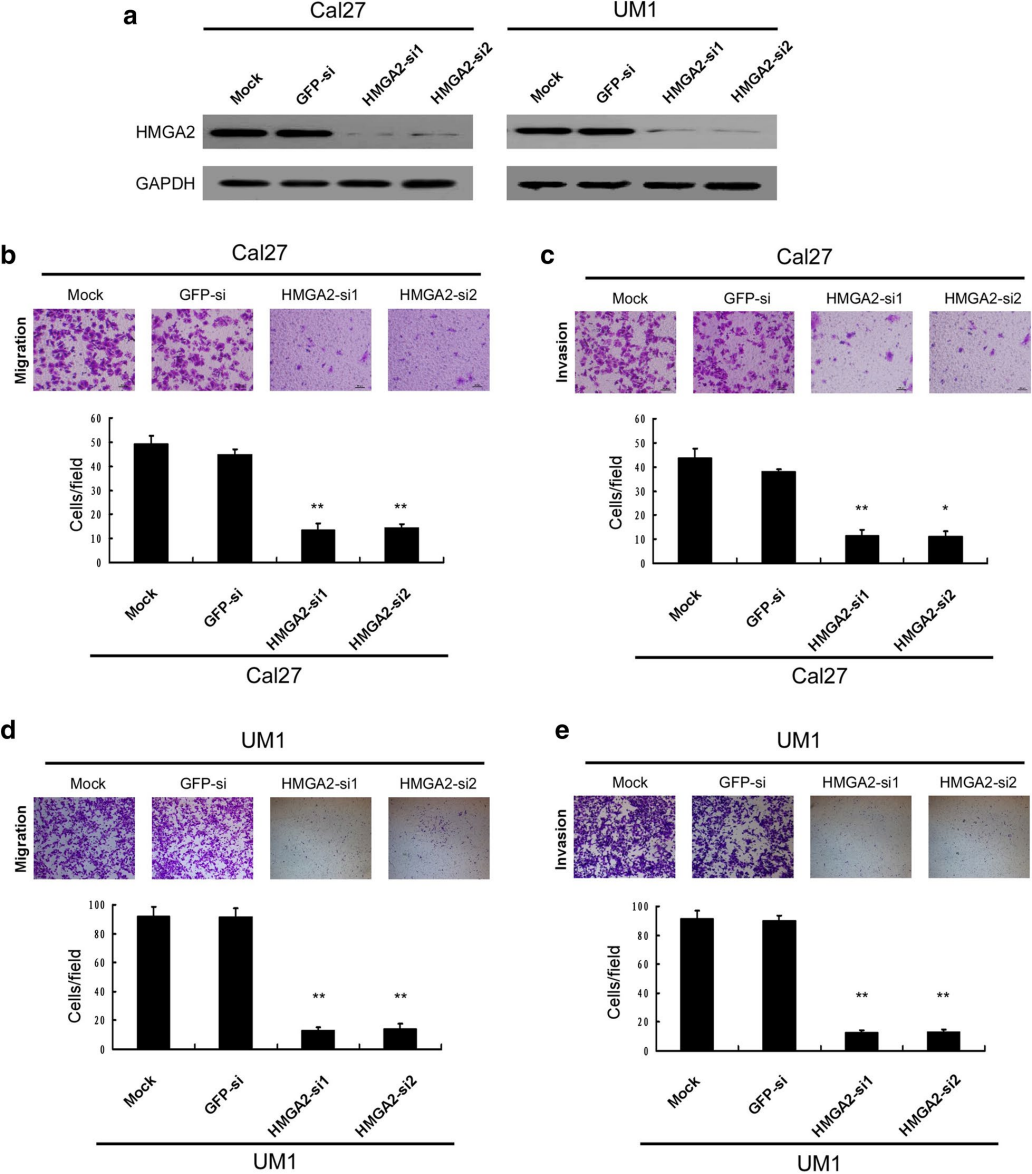
Down-regulation of HMGA2 inhibited TSCC cell motility and invasion. **a** The knockdown efficiency of two specific siRNA against HMGA2 was examined by Western blotting in Cal27 and UM1 cells. **b** The migration and **c** invasiveness abilities were analyzed in an epithelial type tongue cancer cell Cal27 by Boyden chamber assay (*scale bar*: 200 μm, * *P* < 0.05, ** *P* < 0.01). **d** The migration and **e** invasiveness abilities were analyzed in a mesenchymal like tongue cancer cell UM1 by Boyden chamber assay (*scale bar*: 200 μm, ** *P* < 0.01)

As HMGA2 expression was positively related with lymph node metastasis and metastasis to distant organs contributes to poorer survival, next we investigate the effect of HMGA2 on the migration and invasion of Cal27 epithelial tongue cancer cell and UM1 mesenchymal tongue cancer cell. Firstly, the cell lines were treated with two specific siRNAs against HMGA2 and the silence efficiency was examined by western blotting in these two cell lines. As shown in Fig. 4a, both siRNAs efficiently knocked down the endogenous expression of HMGA2 protein in Cal27 and UM1 cells. Using the transwell chamber model, we observed that compared to untreated or vector transfecting tongue cancer cells, silencing of HMGA2 expression severely inhibited the migration and invasion ability of Cal27 and UM1cells (Fig. 4b–e). Similar results were also observed in wound healing assay (data not shown). These results indicated that the expression of HMGA2 promotes the metastatic ability of tongue cancer cells.

### HMGA2 promotes EMT phenotype in tongue cancer cells

**Fig. 5:**
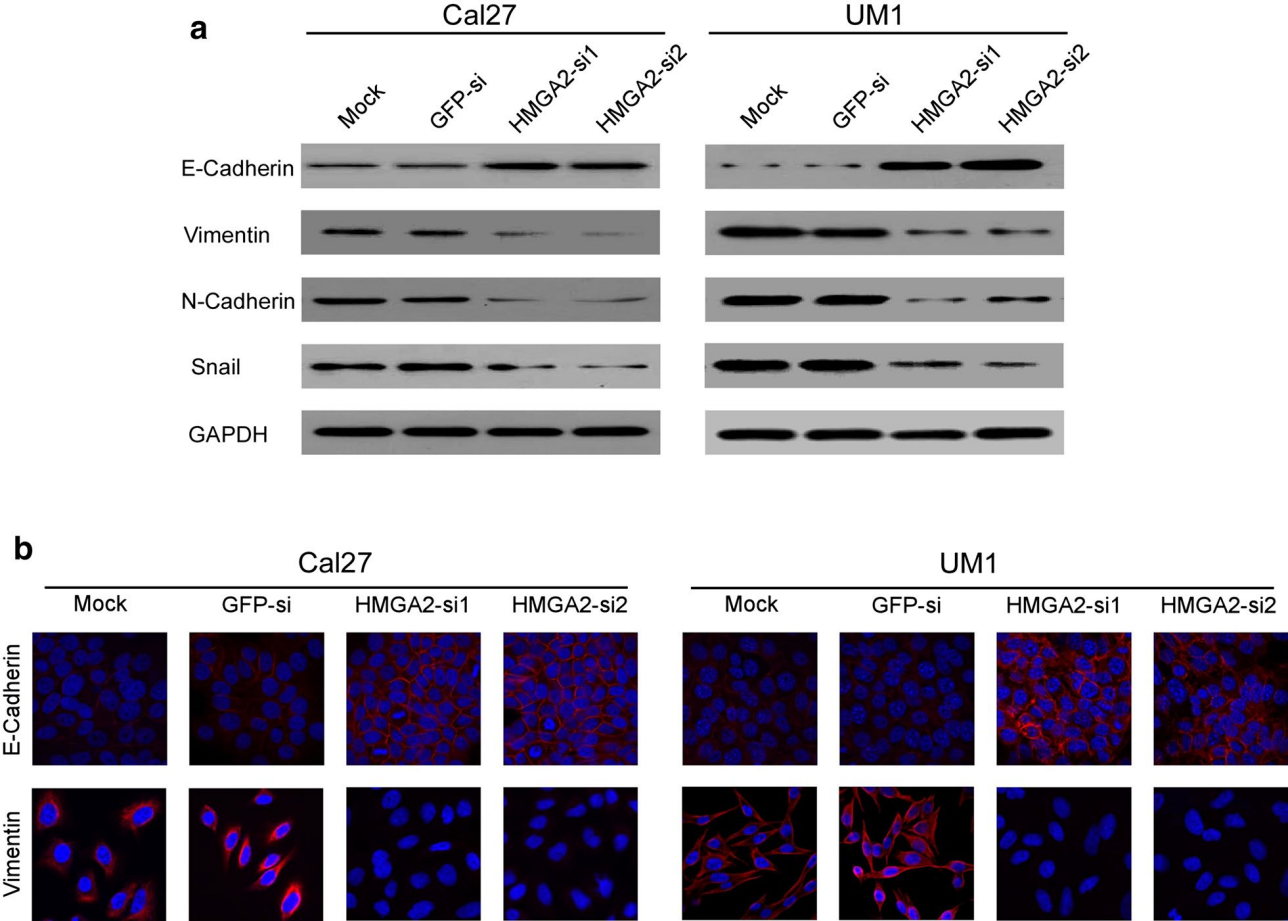
Knockdown of HMGA2 can reversed epithelial–mesenchymal transition (EMT). **a** The expression of EMT markers of E-Cadherin, Vimentin, N-Cadherin and Snail were analyzed by western blotting both in Cal27 and UM1 tongue cancer cells. GAPDH was probed as the loading control. **b** Immunofluorescence staining analysis EMT markers of E-Cadherin and Vimentin (*red*) using confocal and the nuclei were stained with DAPI (*blue*) (*scale bar*: 5 μm)

The results above demonstrate that overexpression of HMGA2 is associated with poorer survival in patients with tongue cancer; however, the mechanism and factors downstream of HMGA2 which mediate this effect are unknown. It has been reported that HMGA2 can induce and promote several cancer cells EMT by up-regulating Snail expression [26]. Furthermore, activation of the EMT is a universal phenomenon in multiple types of cancer [27]. Then, western blotting and immunofluorescence (IF) were performed to investigate the expression of markers of the EMT in tongue cancer cells. We found that in both Cal27 epithelial tongue cancer cells and UM1 mesenchymal tongue cancer cells, silencing of HMGA2 inhibited the expression of Snail and two classical mesenchymal cell markers, N-cadherin and vimentin, whereas the epithelial cell marker, E-cadherin expression was elevated, indicating that there is a potential correlation between HMGA2 and Snail (Fig. 5a). The similar results can be found by IF analysis in both Cal27 and UM1 tongue cancer cells (Fig. 5b). These results imply that the expression of HMGA2 may promote metastasis in tongue cancer by activating the EMT.

### Snail is potential involved in tongue cancer EMT activated by HMGA2

**Fig. 6:**
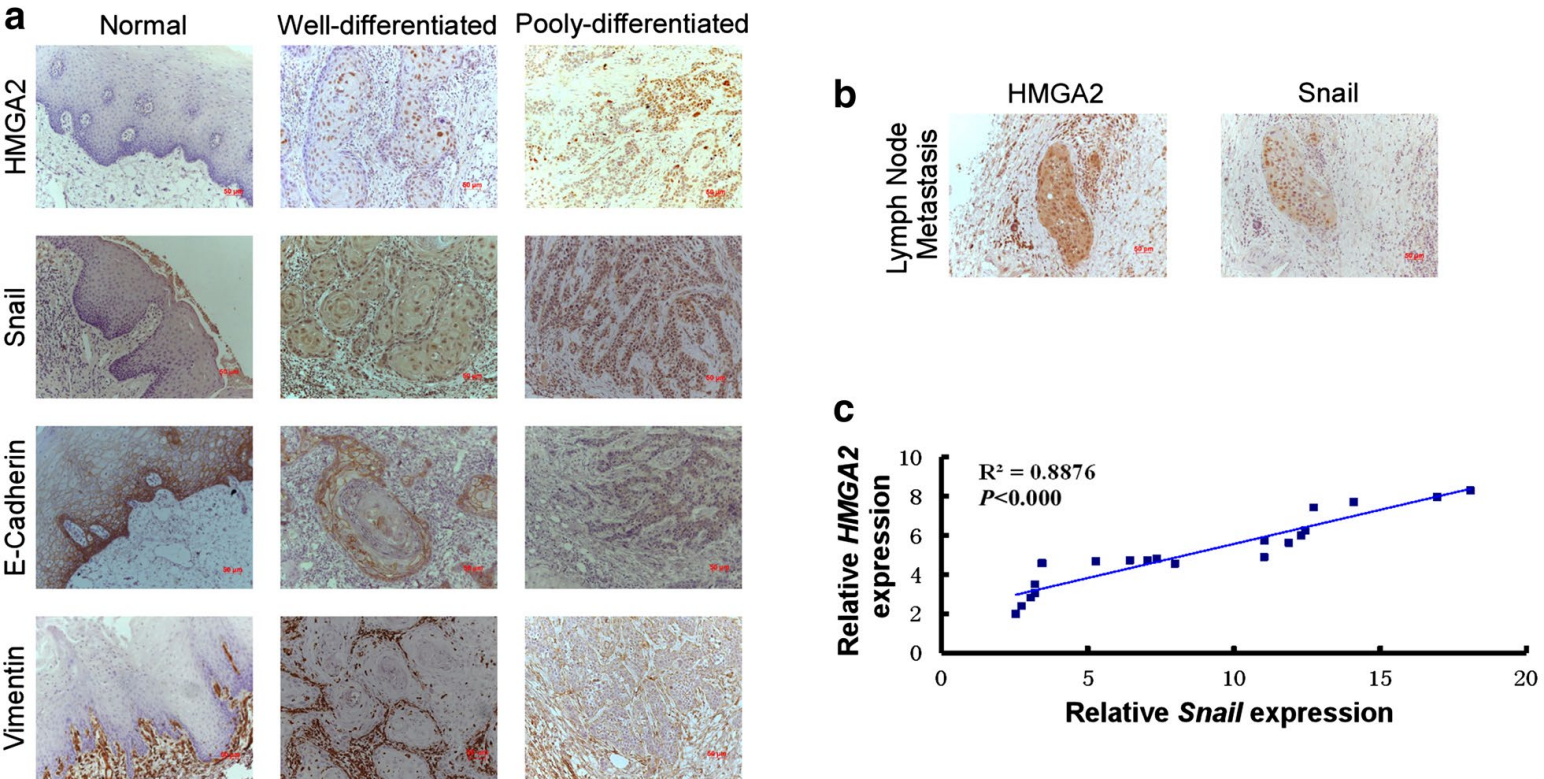
Overexpression of HMGA2 promote TSCC epithelial–mesenchymal transition (EMT) by upregulating Snail. **a** Immunohistochemitry analysis detecting the expression of HMGA2 and EMT markers (E-Cadherin, Vimentin and Snail) in abnormal tongue tissues and primary tongue tumor tissues with different histological differentiation. **b** Immunohistochemical staining showing the high expression and co-localization of HMGA2 and Snail in metastatic lymph node. **c** The correlation analysis showing the positive correlation between HMGA2 and Snail in TSCC patients

Previous reports have revealed that Snail was an important transcriptional factor which can directly induce EMT in multiple cancers [28, 29]. Our data indicated that Snail is potentially involved in the EMT induction by interaction with HMGA2 in tongue cancer. To further demonstrate the correlation between HMGA2 and Snail during the EMT process in tongue cancer, the expression of HMGA2, Snail and EMT markers, E-cadherin and Vimentin were examined in different differential stage tongue cancer tissues. As Fig. 6a shown, the poorly-differentiated tongue cancer tissue expressed much higher levels of HMGA2, Snail and Vimentin, but lower level of E-cadherin, than the well differentiated tongue cancer tissue, compared to the normal tongue tissues (Fig. 6a). As tongue cancer cells preferred to transfer to lymph nodes, we examined the expression of HMGA2 and Snail in the metastatic lymph nodes [30, 31]. We found that both HMGA2 and Snail are highly expressed and co-localized in the nuclear of lymph nodes (Fig. 6b), indicating that there may be an interaction between HMGA2 and Snail, which may play an significant role in the metastasis process through EMT pathway activated by HMGA2 in tongue cancer.

To further illustrate the relationship between HMGA2 and Snail, the correlation between HMGA2 and Snail were analyzed by Person analysis and the result indicated that there is a positive correlation between them (*R*^2^ = 0.8876, *P* < 0.000, Fig. 6c). The clinicopathological and prognostic significance of Snail in tongue cancer is also analyzed by immunohistochemical staining. As shown in Table 1, Snail expression strongly correlated with clinical stage (*P* = 0.003), lymph node metastasis (*P* = 0.000), histological differentiation (*P* = 0.001) and survival (*P* = 0.001). However, multivariate survival analysis revealed that Snail expression was not an independent prognostic factor (*P* = 0.97), whereas HMGA2 was (*P* = 0.042) (Table 2). Collectively, these findings indicate that HMGA2 protein expression, but not Snail protein expression, correlates significantly with the prognosis of patients with tongue cancer.

## Discussion

TSCC is a common and considerable threat to human health in the worldwide. Many researchers have explored the underlying mechanisms which may regulate cancer cell progression in TSCC. It is believed that metastasis is an essential feature of cancer and contributes to the majority of cancer-related deaths in humans and several signal pathways are involved in this procession, including EMT [27, 32, 33]. Epithelial–mesenchymal transition (EMT) is a process whereby tumor cells lose the epithelial features to acquire a mesenchymal phenotype and become motile and invasive, which is closely associated with metastasis [27, 34].

It has been reported that tumor cells can dedifferentiate to obtain the capability to migrate and invade, endowing cancer cells to disseminate from the primary tumor to distant organs, via triggering specific genes expression which associated with EMT signal pathway. Meanwhile, EMT is closely regulated by several signal pathways and involves regulation networks of transcription factors, such as Snail, ZEB and Twist family which regulate expression of E-cadherin, which is a major suppressor of tumor invasiveness and transcriptionally repressed during the EMT [35–37].

HMGA2 is one of the members of the high-mobility group A (HMGA) family which binds to DNA sequences to orchestrate transcription activity by modulating chromatin structure. Besides, HMGA2 is frequently highly expressed in undifferentiated cells during embryogenesis, but silenced in most of the normal adult tissues [21, 38]. So, HMGA2 rarely can be detected in normal adult tissues but is usually reactivated in a variety of benign and malignant tumors. Furthermore, highly expression of HMGA2 has been correlated with cancer proliferation, increased metastasis and poor prognosis in multiple types of cancer [4].

It has been described that up-regulation of HMGA2 can activate the Snail, Twist and ZEB families expression and induce EMT process, which leads to tumor metastasis in various cancers [14]. In this study, our results are consistent with numerous prior studies that HMGA2 is up-regulated both in TSCC cell lines and tissues; the high level expression of HMGA2 can activate the EMT process by repressing E-cadherin expression and the up-regulating of HMGA2 is closely associated with metastasis and poor prognosis in tongue squamous cell carcinoma. Meanwhile, previous researches have implied that Smad, TGF-β canonical pathway and NF-κB signal pathway also contribute to EMT procession through associating with HMGA2 [26, 39, 40]. We show that the overexpression of HMGA2 can up-regulate Snail expression level and activate EMT, leading to poor clinical stage (*P* = 0.001), lymph node status (*P* = 0.000), poor histological differentiation (*P* = 0.002) and short survival (*P* = 0.000) in patients with tongue cancer. Interestingly, although Snail play a pivotal role in the regulating of EMT, multivariate survival analysis shown that Snail expression was not an independent prognostic factor (*P* = 0.97), whereas HMGA2 was (*P* = 0.042), implying that HMGA2 may be an independent prognosis biomarker in the tongue squamous cell carcinoma.

MicroRNA can regulate gene expression by binding to the 3′-untranslational region (3′-UTR) to degrade the target genes expression. In previous studies, HMGA2 was identified as the target gene of several microRNAs, such as *Let*-*7*, which is considered to be a tumor suppressor gene in multiple types of cancer [41–44]. Several researches have revealed a new function and mechanism of HMGA2 as a competing endogenous to promote lung cancer progression [17, 45].

Lymph node metastasis is the predominant invasive site of TSCC and predicted a poor prognosis [31]. Our results show that overexpression of HMGA2 is closely associated with lymph node metastasis and immunohistochemical staining indicate that both HMGA2 and Snail are upregulated and co-localized in the nuclear. Correlation analysis also confirms that there is a positive correlation between them, implying the promoting cooperation during the TSCC progression.

In summary, our study demonstrated that HMGA2 was upregulated and positively associated with the overall survival, clinical stage, T classification and N classification. Moreover, HMGA2 expression is positively correlated with Snail expression in TSCC patients, implying the interaction between each other. In addition, knockdown of HMGA2 expression can severely impair tongue cancer cells migration, invasion and EMT process. This study suggests that HMGA2 may play a pivotal role in tumor metastasis and can be a novel diagnostic marker and potential therapeutic target in TSCC.

## Conclusions

In sum, overexpression of HMGA2 promotes tongue cancer cell migration and invasion in vitro. In addition, up-regulation of HMGA2 was closely associated with poor prognosis in tongue cancer patients. HMGA2 enhances tongue cancer metastasis and progression via interaction with Snail through EMT signal pathway. Together, our findings suggest that HMGA2 participates in the progression of TSCC via Snail through EMT.
